# Echocardiographic Assessment Before, During, and After Impella Positioning: State of the Art

**DOI:** 10.3390/jcm15062404

**Published:** 2026-03-21

**Authors:** Marta Bandini, Alberto Piermartiri, Gioel Gabrio Secco, Edoardo Elia, Rachele Contri, Alina Gallo, Andrea Audo, Giulia Maj

**Affiliations:** 1 Cardiothoracic Intensive Care, Royal Papworth Hospital, Cambridge CB2 0AY, UK; bandinimarta91@gmail.com; 2 Department of Cardiology, University of Bologna, 40126 Bologna, Italy; piermartirialberto@gmail.com; 3 Department of Cardiology, SS. Antonio e Biagio e Cesare Arrigo University Hospital, 15121 Alessandria, Italy; gioel.secco@ospedale.al.it (G.G.S.); edoardo.elia93@gmail.com (E.E.); 4 Department of Cardiothoracic and Vascular Anesthesia and Intensive Care, SS. Antonio e Biagio e Cesare Arrigo University Hospital, 15121 Alessandria, Italy; rachele.contri@gmail.com; 5 Division of Cardiac Surgery, SS. Antonio e Biagio e Cesare Arrigo University Hospital, 15121 Alessandria, Italy; alina.gallo@ospedale.al.it (A.G.); aaudo@ospedale.al.it (A.A.)

**Keywords:** echocardiography, Impella, percutaneous left ventricular assist device, procedural echocardiography guidance, cardiogenic shock, mechanical circulatory support, transesophageal echocardiography, weaning, hemodynamic monitoring

## Abstract

Echocardiographic assessment is essential for evaluating patients with cardiogenic shock (CS) and determining their potential need for mechanical circulatory support (MCS) implantation. The use of Impella devices has increased significantly in recent years, paralleling the growing recognition of their hemodynamic benefits in selected patient populations. As the clinical experience with these devices has expanded, the need for a more standardized imaging approach has emerged. Both transthoracic echocardiography (TTE) and transesophageal echocardiography (TEE) play complementary roles in guiding the pre-implantation evaluation, placement procedure, and post-implantation management of Impella devices. Currently, no comprehensive guidelines exist concerning the echocardiographic evaluation of Impella devices throughout their entire clinical course, from initial patient selection and device implantation to ongoing monitoring and eventual weaning. This gap in standardized guidance has led to significant variability in clinical practice across different institutions and healthcare systems. This comprehensive review examines the role of transthoracic echocardiography (TTE) and transesophageal echocardiography (TEE) in managing patients on Impella support across five distinct phases: candidate identification and pre-implantation assessment, intraoperative procedural guidance and device positioning, postoperative monitoring and haemodynamic optimisation, complication detection and troubleshooting, and weaning strategies with post-explantation surveillance. Both left-sided devices (Impella CP, CP Smart Assist, and Impella 5.5) and right-sided support (Impella RP) are covered, including combined configurations with VA-ECMO (ECPella). For each phase, we detail the recommended echocardiographic views, essential measurements and their evidence-based thresholds, signs of device malposition, and practical corrective strategies. A level-of-evidence approach is adopted throughout, specifying whether proposed thresholds derive from randomised trials, observational studies, expert consensus, or manufacturer recommendations. Summary tables and a bedside workflow are provided to facilitate immediate clinical application.

## 1. Introduction

Impella devices are percutaneous microaxial flow pumps that have become increasingly utilized in the management of cardiogenic shock (CS) and high-risk cardiac interventions over the past two decades. The Impella device (Abiomed, Danvers, MA, USA) is an intravascular microaxial pump that provides temporary mechanical support to patients by actively unloading the ventricle, thereby reducing the heart’s workload while simultaneously improving systemic circulation. Since its introduction into clinical practice, the Impella has become an integral tool in the armamentarium of clinicians caring for critically ill patients with acute cardiac decompensation [1].

The pathophysiological rationale for mechanical ventricular unloading is well established. In cardiogenic shock, the failing ventricle is trapped in a vicious cycle of inadequate cardiac output, compensatory neurohormonal activation, increased wall stress, and progressive myocardial injury. By actively aspirating blood from the ventricle and propelling it into the arterial system, Impella devices interrupt this cycle by reducing ventricular filling pressures, decreasing wall stress, and improving coronary perfusion pressure. These hemodynamic effects create a more favorable environment for myocardial recovery while maintaining adequate systemic perfusion [2].

Over recent years, the clinical indications for Impella therapy have expanded considerably, and the development of standardized criteria for patient selection, imaging guidance, and device monitoring has become essential for optimizing outcomes. The DanGer Shock trial—a multicentre, randomised, open-label superiority trial enrolling 355 patients—reported a statistically significant reduction in 180-day all-cause mortality (relative risk 0.74, 95% CI 0.55–0.99; absolute risk reduction 8.0 percentage points) with early Impella CP implantation in patients with AMI-CS, providing the first Level A evidence supporting the role of these devices in this population [1]. Notably, this trial was limited to Impella CP in AMI-related cardiogenic shock; extrapolation of these results to the Impella 5.5, to non-ischaemic aetiologies, or to the Impella RP should be made with caution pending further dedicated studies [1]. Of particular relevance to this review, the DanGer Shock trial protocol relied on echocardiographic assessment to confirm left ventricular dysfunction as the primary cause of shock and to exclude mechanical complications (ventricular septal defect, free wall rupture, acute severe mitral regurgitation) as well as predominant right ventricular failure as the main haemodynamic driver. These echocardiographic criteria served as a gatekeeper for appropriate patient selection and provide a practical reference for clinicians evaluating Impella candidacy in the acute setting, reinforcing the central message of this review: systematic echocardiographic evaluation is essential before initiating mechanical circulatory support.

However, the success of Impella therapy depends critically on appropriate patient selection, accurate device positioning, and vigilant monitoring throughout the support period. The technical characteristics of the various Impella devices differ substantially in terms of catheter size, flow capacity, insertion route, and duration of support. These differences have important implications for patient selection, insertion technique, and monitoring strategies. The characteristics of currently available Impella devices are summarized in Table 1.

Left-sided Impella systems encompass two distinct categories that differ in catheter calibre, insertion route, flow capacity, and approved duration of support. The Impella CP and Impella CP Smart Assist are lower-profile devices (9 Fr catheter shaft/14 Fr pump motor) designed for percutaneous femoral arterial insertion; they provide up to 3.5–4.0 L/min of forward flow and are approved for short-term support (up to 4–6 days according to the manufacturer’s instructions for use). The Impella 5.5 is a larger-bore device (9 Fr shaft/19 Fr pump motor) that requires surgical vascular access—typically via axillary or subclavian arteriotomy—and delivers up to 5.5 L/min of flow, with an approved support duration of up to 14 days. This surgical access route has important implications for patient selection, procedural planning, and the echocardiographic views used for device monitoring, as the catheter trajectory through the aortic arch differs from the femoral approach. All left-sided Impella devices are positioned retrogradely across the aortic valve into the left ventricle (LV), where they aspirate blood through an inlet positioned near the ventricular apex and deliver forward flow into the ascending aorta through an outlet positioned above the aortic valve. This configuration effectively reduces LV end-diastolic pressure and wall stress, enhances coronary perfusion by increasing aortic root diastolic pressure, and decreases myocardial oxygen demand by reducing ventricular work. The hemodynamic benefits of left ventricular unloading extend beyond simple augmentation of cardiac output to include favorable effects on myocardial energetics and recovery potential [3].

The right-sided Impella RP addresses the distinct challenge of right ventricular failure, which carries a particularly poor prognosis when it complicates cardiogenic shock. The Impella RP is introduced via the femoral vein and advanced across the tricuspid and pulmonary valves to provide up to 4.0 L/min of pulmonary arterial flow, thereby unloading the right ventricle (RV). Specifically, the Impella RP is a 22 Fr, 22-cm-length microaxial flow pump device that is percutaneously implanted through a 23 Fr peel-away sheath inserted into the femoral vein and positioned with its inlet in the inferior vena cava and its outlet in the pulmonary artery [3]. Unlike the left-sided Impella 5.5, which requires surgical vascular access, the Impella RP is a fully percutaneous device; however, its large venous sheath size (23 Fr) necessitates careful pre-procedural assessment of femoral venous calibre and patency. The pump operates at speeds delivering up to 4.0 L/min and may remain implanted for up to 14 days, making it the longest-approved percutaneous Impella configuration currently available for right ventricular support. The more recently introduced Impella RP Flex—a repositionable iteration with a smaller access profile—may further expand the population eligible for percutaneous right-sided support, although clinical experience with this device is still limited [4].

**Figure 1:**
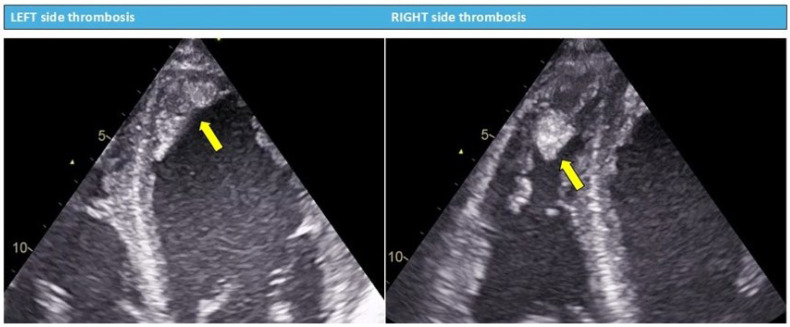
Electrocardiographic characteristics of ventricular thrombosis. Yellow arrows: echo-dense masses consistent with ventricular thrombi, with well-defined borders distinct from the endocardium, visible throughout systole and diastole.

The distinction between percutaneous and surgical access strategies has practical implications for echocardiographic assessment that should be recognised. Percutaneous femoral insertion (Impella CP, CP Smart Assist, Impella RP) allows rapid deployment—including at the bedside or in the catheterisation laboratory—and is typically guided by a combination of fluoroscopy and echocardiography. Surgical insertion (Impella 5.5 via axillary or subclavian arteriotomy) is performed in the operating room or hybrid suite and may be associated with a different catheter trajectory through the aortic arch, potentially affecting the echocardiographic windows best suited for assessing device position. Furthermore, surgically implanted devices are generally intended for longer support durations, which increases the importance of serial echocardiographic surveillance for delayed complications such as device migration, progressive valvular injury, and thrombus formation. These differences are summarised in Table 1 and illustrated schematically in Figure 1, which provides an overview of all Impella device types and configurations covered in this review, including access route, catheter size, flow capacity, approved duration of support, and anatomical positioning. These distinctions should be taken into account when planning the imaging follow-up strategy for each individual patient.

As Impella systems are now employed across an increasingly wide range of clinical indications, including acute myocardial infarction with cardiogenic shock, decompensated heart failure, high-risk percutaneous coronary intervention, post-cardiotomy shock, and acute myocarditis, it has become essential to establish clear criteria for patient candidacy to demonstrate the true impact of the device on patient outcomes. Moreover, correct device positioning and meticulous ongoing management are crucial to ensure the desired hemodynamic support without introducing iatrogenic complications [5]. The consequences of suboptimal device positioning include inadequate ventricular unloading, hemolysis, valvular injury, and thromboembolic events—all of which can significantly worsen patient outcomes.

Echocardiography has emerged as the cornerstone imaging modality in this context, serving essential roles in patient selection, procedural guidance, and ongoing device management. The real-time visualization capabilities of echocardiography, combined with its ability to assess cardiac structure, function, and hemodynamics non-invasively, make it ideally suited for all phases of Impella therapy. This comprehensive review examines the role of transesophageal echocardiography (TEE) and transthoracic echocardiography (TTE) in managing patients on Impella support at different phases, including candidate identification, device insertion, maintenance and optimization, complication detection and management, and eventual weaning and removal.

## 2. Methods

This narrative review was conducted following a structured literature search aimed at synthesizing the current evidence on the echocardiographic assessment of Impella percutaneous ventricular assist devices across all phases of clinical management. A comprehensive search of the PubMed/MEDLINE, Embase, and Cochrane Library databases was performed from inception through January 2025, using the following search terms and their combinations: “Impella”, “percutaneous ventricular assist device”, “microaxial pump”, “echocardiography”, “transesophageal echocardiography”, “transthoracic echocardiography”, “cardiogenic shock”, “mechanical circulatory support”, “device positioning”, “weaning”, and “hemodynamic monitoring”. Boolean operators (AND, OR) were employed to optimize search sensitivity and specificity. The search was limited to articles published in the English language. Original research articles, case series, case reports, prior systematic and narrative reviews, editorials, expert consensus documents, and clinical practice guidelines were considered eligible for inclusion. Studies were selected based on their relevance to the echocardiographic evaluation of Impella devices in at least one of the following domains: pre-implantation patient assessment and candidate selection, intraoperative procedural guidance and device positioning, postoperative monitoring and complication detection, and weaning strategies and post-explantation surveillance. No restrictions were applied regarding study design, sample size, or patient population. The reference lists of all identified articles were manually screened to retrieve additional relevant publications not captured by the electronic database search. Manufacturer-provided technical documentation and device-specific instructions for use were also consulted to supplement the clinical evidence base. Two reviewers independently screened titles and abstracts for eligibility, with discrepancies resolved by consensus. Given the heterogeneity of the available literature and the predominantly descriptive nature of the included studies, a formal systematic review methodology with meta-analytic pooling was not deemed appropriate. Instead, the findings were synthesized qualitatively and organized according to the temporal phases of Impella therapy to provide a practical, clinically oriented framework for echocardiographic assessment. This review was not registered in any prospective review database (e.g., PROSPERO) and did not receive ethical approval, as it did not involve primary data collection from human subjects.

## 3. Patient Selection and Preoperative Evaluation

The success of Impella therapy begins with careful patient selection and thorough preoperative evaluation. Echocardiography plays a central role in this process by identifying both indications for mechanical support and potential contraindications that might preclude safe device implantation. A systematic echocardiographic assessment allows clinicians to characterize the severity and etiology of cardiac dysfunction, evaluate the suitability of cardiac anatomy for device placement, and anticipate potential challenges during the insertion procedure.

### 3.1. Cardiogenic Shock

Cardiogenic shock (CS) remains one of the most challenging conditions in cardiovascular medicine, associated with in-hospital and short-term mortality rates reported in the range of 40–50% in contemporary registries and clinical trials [6,7]. The Society for Cardiovascular Angiography and Interventions (SCAI) classification system has provided a standardized framework for characterizing the severity of cardiogenic shock and guiding therapeutic decision-making. According to recent evidence from the DanGer Shock trial and other studies, the Impella device plays a significant role in patients presenting with SCAI stages C (classic cardiogenic shock) and D (deteriorating), where early mechanical unloading may improve outcomes [1].

In this clinical setting, it is crucial to rule out any contraindications before proceeding with device implantation. Transesophageal echocardiography (TEE) represents a reliable and highly sensitive modality for identifying potentially reversible causes of cardiogenic shock that might require alternative or additional interventions. These include cardiac tamponade, which requires urgent pericardiocentesis or surgical drainage; papillary muscle rupture with acute severe mitral regurgitation, which typically requires emergent surgical repair; ventricular septal defect complicating myocardial infarction, which necessitates surgical or percutaneous closure; and acute aortic dissection, which may require emergency surgical intervention. Additionally, TEE is invaluable for assessing anatomical contraindications to Impella placement [8].

The aortic valve should be the first structure carefully evaluated during the preoperative echocardiographic assessment. Using the mid-esophageal aortic valve long-axis (LAX) and short-axis (SAX) views, the echocardiographer must assess valve morphology, leaflet mobility, and the presence of regurgitation or stenosis. The presence of moderate-to-severe aortic regurgitation is considered an absolute contraindication to left-sided Impella placement according to the device manufacturer’s instructions for use and current expert consensus, as the device would create a circular shunt with blood flowing retrograde through the incompetent valve while simultaneously being pumped antegrade by the device. Similarly, severe aortic stenosis is generally regarded as precluding device insertion due to the inability to safely advance the catheter across the calcified, stenotic valve and the risk of acute hemodynamic decompensation. A mechanical aortic prosthesis is also listed as an absolute contraindication in the manufacturer’s labelling due to the risk of mechanical interference with prosthetic leaflet function and potential catastrophic valve dysfunction.

A comprehensive assessment of biventricular function is essential for characterizing global ventricular contractility and determining the appropriateness of mechanical support. This assessment should include quantification of ejection fraction, measurement of chamber diameters and volumes, and evaluation of regional wall motion abnormalities. The mid-esophageal four-chamber and two-chamber views provide excellent visualization of the left ventricle for these measurements, while the transgastric short-axis view allows assessment of circumferential contraction and identification of regional dysfunction corresponding to specific coronary territories.

Particular attention should be paid to right ventricular (RV) function during the pre-implantation assessment. Key parameters include TAPSE (concerning if <17 mm), tissue Doppler S′ velocity at the lateral tricuspid annulus (concerning if <9.5 cm/s), RV fractional area change (RVFAC; concerning if <35%), and the RV/LV basal diameter ratio (abnormal if >1.0), as defined by the 2015 ASE/EACVI chamber quantification guidelines (Lang et al. [9]). The presence of moderate-to-severe RV dilatation with systolic dysfunction, significant tricuspid regurgitation, and septal flattening (eccentricity index > 1.1) suggests concomitant RV failure. In this setting, isolated left-sided Impella support may be insufficient and could precipitate haemodynamic deterioration by increasing LV output against a failing right ventricle; biventricular support (e.g., Impella CP + Impella RP, or alternative configurations) or VA-ECMO should be considered. The hemodynamic effects of left ventricular unloading include increased venous return to the right heart, which a severely dysfunctional right ventricle may be unable to accommodate. This can precipitate acute right ventricular failure and hemodynamic collapse despite adequate left ventricular support. The RV should therefore be carefully examined in the mid-esophageal RV inflow-outflow and four-chamber views to characterize its size, morphology, and contractile function.

Evaluating RV performance remains one of the most challenging aspects of clinical echocardiography due to the complex geometry of the right ventricle. A multiparametric approach, as recommended by the ESC/EACVI guidelines, should be adopted to overcome the limitations of any single measurement [10]. This comprehensive assessment should include evaluation of RV morphology and size, with particular attention to the RV/LV basal diameter ratio (abnormal if >1.0 per ASE/EACVI chamber quantification guidelines). The presence of septal flattening, indicating elevated RV pressures, should be assessed with calculation of the left ventricular eccentricity index (abnormal if >1.1, a threshold based on early observational studies by Ryan et al. and incorporated into expert consensus documents [11].

The quantification of longitudinal function using tricuspid annular plane systolic excursion TAPSE (normal > 17 mm) and tissue Doppler S′ velocity at the lateral tricuspid annulus (normal > 9.5 cm/s), and the RV fractional area change (normal > 35%). These thresholds are derived from the 2015 ASE/EACVI guidelines for cardiac chamber quantification and represent population-based normative values; their predictive accuracy for clinical decision-making in the specific context of Impella candidacy has not been prospectively validated [9].

Before proceeding with Impella implantation, the presence of ventricular thrombus should be carefully excluded, as it is considered a well-recognised contraindication to device placement in the manufacturer’s instructions for use and in published expert recommendations [7,11].

The passage of the guidewire and catheter through a thrombus-containing ventricle carries an unacceptable risk of thromboembolism, potentially resulting in stroke, limb ischemia, or other devastating complications. Conversely, in patients with severe LV dysfunction who develop progressive ventricular distension, TEE evaluation through the mid-esophageal four-chamber, long-axis, and transgastric short-axis views is strongly recommended by expert opinion for detecting blood stasis within the LV cavity (manifesting as spontaneous echo contrast or “smoke”) and documenting the absence of aortic valve opening—findings that support the timely decision to initiate mechanical ventricular unloading before irreversible myocardial injury occurs [8,9].

**Figure 2:**
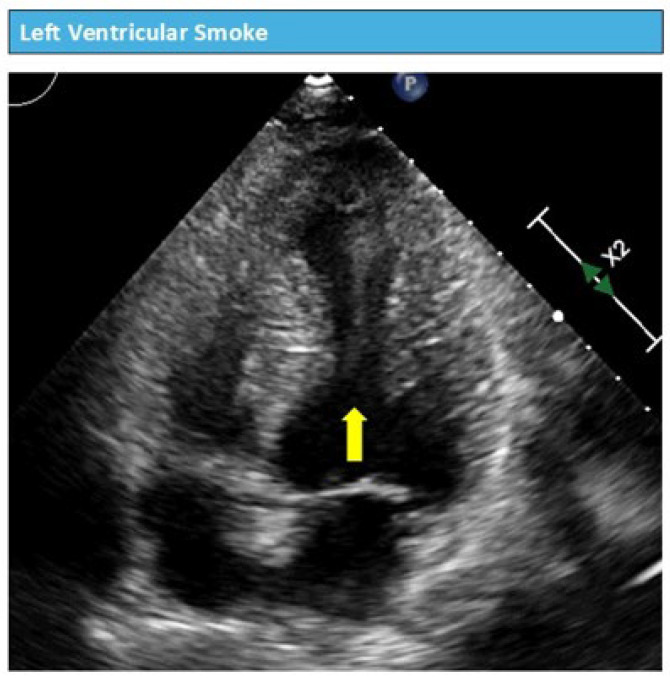
Echocardiographic evaluation of Blood stasis. Spontaneous echocardiographic contrast (smoke) is caused by increased ultrasound backscatter due to the agglomeration of cellular blood components in low-flow states. Yellow arrow: spontaneous echocardiographic contrast (‘smoke’) within the left ventricular cavity, indicative of blood stasis and increased thromboembolic risk.

A preliminary transthoracic echocardiogram may be helpful in selected cases, particularly when the clinical situation permits, to rule out overt ventricular thrombosis or to document the presence of severe spontaneous echo contrast (the “smoke-like effect”), as demonstrated in Figure 1 and Figure 2. TTE has the advantage of being less invasive than TEE and can be performed rapidly at the bedside without the need for sedation or airway protection.

Finally, the ascending and descending thoracic aorta should be systematically evaluated to exclude aortic pathology that might complicate or contraindicate device insertion. The mid-esophageal ascending aortic long-axis view, along with the descending aortic SAX and LAX projections, should be used to exclude aortic dissection, significant atherosclerotic disease, or complex atheromatous plaque. This assessment is particularly important when femoral arterial access is planned for device insertion, as retrograde catheter passage through a diseased aorta carries risks of plaque disruption, embolization, and vascular injury (Table 2).

### 3.2. ECPella: Combined VA-ECMO and Impella Support

Veno-arterial extracorporeal membrane oxygenation (VA-ECMO) has become an increasingly utilized form of mechanical circulatory support in refractory cardiogenic shock. While VA-ECMO effectively restores systemic perfusion and provides respiratory support, it does so at the expense of significantly increased LV afterload due to the retrograde arterial flow generated by the ECMO circuit. This increased afterload can be particularly detrimental to an already failing left ventricle, leading to progressive LV distension, elevated filling pressures, pulmonary edema, and inadequate myocardial unloading that may impair recovery.

In this challenging clinical setting, the combination of VA-ECMO with an Impella device—a strategy commonly referred to as “ECPELLA”—has emerged as a solution to address the problem of LV distension. By adding Impella support to VA-ECMO, clinicians can achieve direct LV unloading while maintaining the peripheral perfusion and oxygenation benefits of ECMO. The ECPELLA configuration has been shown to effectively reduce pulmonary capillary wedge pressure, improve pulmonary blood flow by reducing right ventricular afterload, and decrease LV dimensions, thereby creating more favorable conditions for myocardial recovery [12].

Transesophageal echocardiography provides detailed and essential information on LV geometry and performance that is critical for guiding the decision to add Impella support to an existing VA-ECMO circuit. Serial echocardiographic assessments are strongly recommended in all patients on VA-ECMO, although achieving accurate and reproducible measurements can be challenging in this critically ill patient population due to factors such as mechanical ventilation, patient positioning, and the presence of multiple intravascular catheters [13].

Among the various echocardiographic parameters, assessments of aortic valve opening and the LV outflow tract (LVOT) velocity–time integral (VTI) may provide the most objective and reproducible markers of native cardiac function during ECMO support. The complete absence of aortic valve opening indicates that the left ventricle is generating insufficient pressure to overcome the combined resistance of the aortic valve and the elevated afterload created by ECMO, suggesting severe LV dysfunction requiring urgent unloading. An LVOT VTI < 10 cm has been proposed as a predictor of insufficient intrinsic cardiac output in a single-centre prospective observational study by Aissaoui et al. (n = 51) [14]. While this threshold is widely cited in the VA-ECMO literature, it has not been externally validated in larger multicentre cohorts and should be interpreted as observational-level evidence (Level C).

Other echocardiographic criteria described in the literature for identifying patients who would benefit from left ventricular unloading include a markedly dilated LV, which has been operationally defined in the ECMO literature as end-diastolic diameter (EDD) > 6.1 cm [15]. This value derives from expert opinion and retrospective case series rather than from prospectively validated diagnostic thresholds, and the presence of severe spontaneous echo contrast or “smoke” within the LV cavity [15].

### 3.3. Right Ventricular Failure

Right ventricular failure represents a distinct and particularly challenging clinical entity characterized by poor outcomes in the setting of cardiogenic shock. The right ventricle, with its thin wall, crescent shape, and dependence on adequate preload and low afterload, is inherently more vulnerable to acute decompensation than the left ventricle. RV failure may occur as a primary cardiomyopathy (such as arrhythmogenic right ventricular cardiomyopathy), as a consequence of acutely increased afterload in the setting of massive pulmonary embolism or decompensated chronic pulmonary hypertension, or in the post-cardiotomy scenario following cardiac surgery.

In contrast to the percutaneous left-sided Impella devices, which can be positioned using either fluoroscopic or echocardiographic guidance, the Impella RP is currently inserted only under fluoroscopic guidance due to the complex trajectory required to navigate the device from the femoral vein through the right atrium, across the tricuspid valve, through the right ventricle, and across the pulmonary valve into the pulmonary artery. However, comprehensive echocardiographic evaluation remains essential for preoperative anatomic and functional RV assessment and to systematically rule out any contraindications to device placement [16].

Moreover, RV failure cannot be adequately evaluated in isolation from the left ventricle. The two ventricles share the interventricular septum, are enclosed within a common pericardium, and are connected in series through the pulmonary circulation. Isolated RV dysfunction and biventricular dysfunction are therefore two completely different clinical conditions with distinct implications for management. Importantly, implantation of an isolated RV assistance device in a patient with unrecognized LV dysfunction can potentially precipitate or worsen LV failure by increasing preload to a compromised left ventricle.

Preoperative echocardiographic evaluation for Impella RP candidates should utilize the mid-esophageal four-chamber view, mid-esophageal right ventricle inflow-outflow view, and trans-gastric right ventricle inflow view to systematically rule out the presence of any absolute contraindications. These contraindications include mechanical tricuspid or pulmonary valve prostheses, which would prevent safe device passage; moderate or severe tricuspid or pulmonary valvular stenosis, which would impede catheter advancement; and mural thrombus in the right atrium or vena cava, which could be dislodged during device insertion, causing pulmonary embolism.

RV failure characteristically presents with contractile dysfunction and RV dilatation and is frequently associated with functional tricuspid regurgitation secondary to annular dilatation. The presence and severity of tricuspid regurgitation poses a particular challenge in this context. Significant tricuspid regurgitation may alter the quantification of RV stroke volume and ejection fraction, complicating the assessment of native RV function. According to the device manufacturer, severe tricuspid regurgitation would represent a relative contraindication to Impella RP placement. However, clinical experience suggests that Impella RP may still effectively unload the RV if the pulmonary valve is competent, and that functional tricuspid regurgitation from annulus dilatation may actually improve due to RV reverse remodeling after successful mechanical unloading. Additionally, pulmonary valve regurgitation is not infrequent in patients with RV dysfunction and increased pulmonary vascular resistance and should be documented during the preoperative assessment [5].

**Figure 3:**
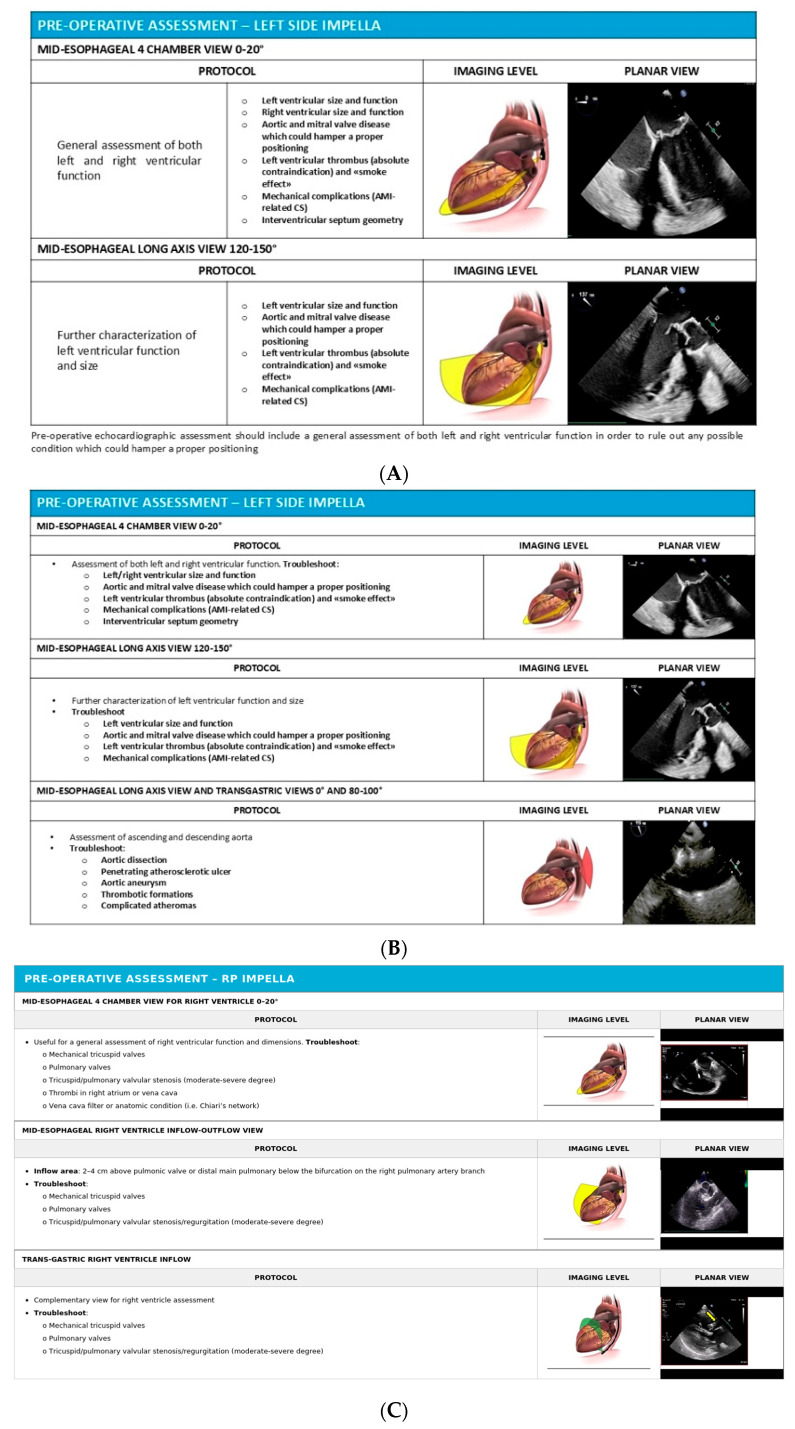
(**A**,**B**) Pre-operative echocardiographic assessment for left-side Impella^®^ positioning. (**C**) Pre-operative echocardiographic assessment for RP Impella^®^ positioning.

The degree of RV dilatation and remodeling should be carefully evaluated, as severe chamber enlargement can make device implantation technically more challenging and increases the risk of subsequent device displacement. In patients with severely dilated and remodeled right ventricles, the Impella RP catheter may have difficulty maintaining optimal positioning, potentially requiring more frequent monitoring and repositioning maneuvers [5,17]. Pre-implantation assessment protocols for both left-sided and right-sided Impella devices are illustrated in Figure 3A–C. The key echocardiographic parameters used for patient selection and indications for each type of mechanical circulatory support are summarised in Table 2.

## 4. Intraoperative Evaluation for Left-Sided Impella

Left-sided Impella placement is typically performed under fluoroscopic guidance. For the percutaneously inserted Impella CP and CP Smart Assist, the procedure is usually carried out in the cardiac catheterisation laboratory, whereas the surgically inserted Impella 5.5 is placed in the operating room or a hybrid suite under direct surgical visualisation of the access vessel. In both settings, transesophageal echocardiography may serve as an acceptable alternative or adjunct imaging modality [18].

In clinical practice, the combination of fluoroscopy and TEE provides the best spatial orientation and allows real-time assessment of both device position and cardiac function. Fluoroscopy provides excellent visualization of the radiopaque device components and their relationship to bony landmarks, while echocardiography adds critical information about soft tissue structures including the valve apparatus, ventricular walls, and myocardial function.

**Figure 4:**
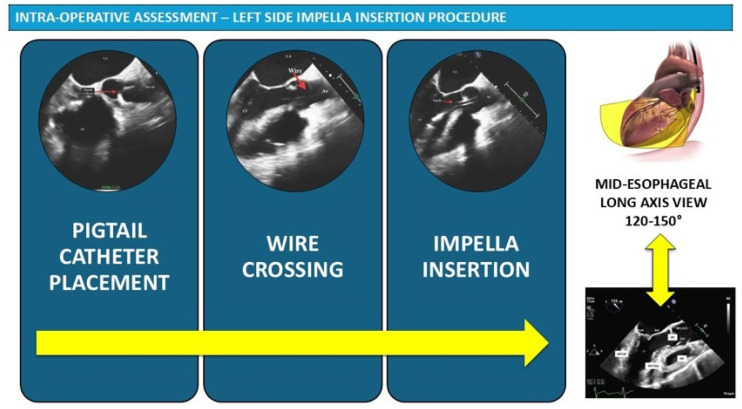
Main views and steps for intra-operative echocardiographic assessment for Impella^®^ positioning. 3-steps approach for left side Impella positioning: the mid-esophageal long-axis view is pivotal in evaluating the proper positioning and the possible interference of the Impella catheter with mitral valves and sub-valvular apparatus.

When TEE is used for procedural guidance, the insertion procedure should be followed primarily through the mid-esophageal long-axis view, which provides excellent visualization of the aortic valve, left ventricular outflow tract, and LV cavity. The initial step involves confirming the presence of the guidewire positioned in the ventricular apex after crossing the aortic valve. It is essential to ensure that the wire is not entangled with papillary muscles or chordae tendinae, as this could result in mitral valve injury during subsequent catheter advancement (Figure 4). The guidewire should course smoothly from the ascending aorta, across the aortic valve, through the LV cavity, and toward the ventricular apex without any acute angulation or looping.

Once the guidewire position is confirmed to be satisfactory, the Impella catheter is advanced over the wire through the femoral sheath and into the ascending aorta. The mid-esophageal aortic valve long-axis view and the mid-esophageal aortic valve SAX view should be used in combination to ensure excellent positioning across the valve. The catheter should cross the aortic valve smoothly without excessive resistance, and the echocardiographer should confirm that the device does not impinge on or injure the aortic valve leaflets during passage.

The optimal final position of a left-sided Impella places the inlet zone (where blood is aspirated) within the left ventricular cavity and the outlet area (where blood is ejected) in the ascending aorta above the aortic valve. The inlet must be directed toward the LV apex and should be positioned between the papillary muscles to avoid obstruction and optimize flow. Color-flow Doppler imaging adds valuable information about the pump inflow and outflow, allowing confirmation of appropriate device function and detection of any associated valvular regurgitation.

Precise positioning requires careful attention to the depth of device insertion across the aortic valve. For the percutaneously inserted Impella CP, the device tip (inlet) should be positioned 3.5–4 cm below the aortic valve plane according to the manufacturer’s instructions for use. For the surgically inserted Impella 5.5, a slightly deeper position of approximately 4–5 cm below the valve is recommended (manufacturer recommendation). The deeper target for the Impella 5.5 reflects the larger pump housing and the different catheter trajectory associated with axillary or subclavian access. These positioning targets are specified in the manufacturer’s instructions for use and supported by expert consensus [6,19]; no randomised studies have compared different insertion depths with respect to clinical outcomes.

It is critically important not to include the pigtail—a curved extension present only on the Impella CP that helps stabilize the device in the ventricle—in this depth measurement. Including the pigtail in the measurement is a common mistake that may lead to erroneous pullback of a correctly positioned device, resulting in inadequate ventricular unloading. Measurements should be obtained through multiple imaging planes because the catheter follows a curved trajectory through the aortic root and into the ventricle. The pump outlet should be confirmed to be located approximately 1.5 cm above the aortic valve plane, as recommended by the device manufacturer. This distance has been adopted by convention to minimise the risk of outlet obstruction and aortic valve interference, but has not been derived from comparative clinical studies.

Another critical aspect of intraoperative echocardiographic assessment during Impella placement is evaluation of the potential interference of the catheter with the mitral and aortic valve apparatus. The mitral subvalvular structures—including the papillary muscles, chordae tendinae, and leaflets—should be carefully evaluated to ensure that the device does not cause mechanical tethering or leaflet restriction that could result in mitral regurgitation or inflow obstruction. If evidence of mitral valve tethering is identified, the Impella must be immediately repositioned to avoid these complications.

Similarly, the aortic valve should be reassessed after device placement using the mid-esophageal long-axis and short-axis views to confirm normal leaflet motion and to exclude device-induced trauma or interference. The occurrence of new or worsening aortic regurgitation following device placement may indicate valve leaflet injury or distortion of the valve annulus and warrants immediate reassessment and potential repositioning of the device.

**Figure 5:**
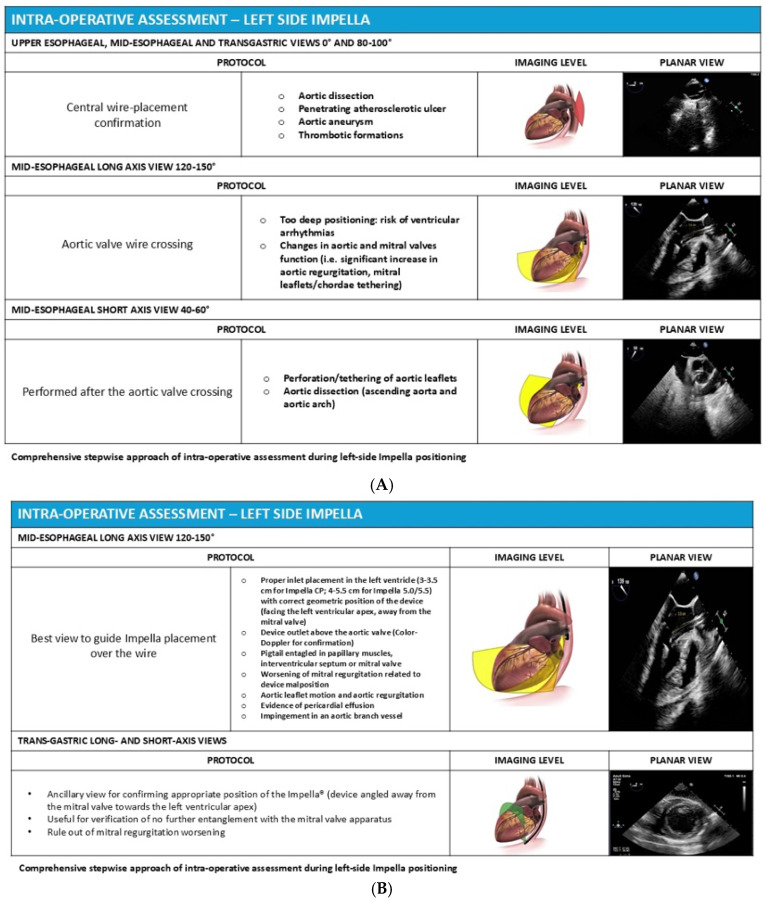
(**A**,**B**) Intra-operative echocardiographic assessment for Impella^®^ positioning.

Once the Impella has been confirmed to be in optimal position, the guidewire is carefully removed under echocardiographic and fluoroscopic observation. A final comprehensive verification should then be performed in the mid-esophageal long-axis view to ensure that the catheter does not contact the interventricular septum (which could cause suction events and hemolysis) or the mitral valve apparatus (which could cause valvular injury or regurgitation). Representative intraoperative echocardiographic views demonstrating optimal left-sided Impella placement are shown in Figure 5A,B and Table 3.

## 5. Intraoperative Evaluation for Impella RP Insertion

The Impella RP device is implanted exclusively under fluoroscopic guidance due to the complex trajectory required to navigate the catheter from the femoral venous access site, through the right heart chambers, and into the pulmonary artery. However, intraoperative echocardiography—whether TEE or TTE—provides crucial complementary information for assessing and optimizing device placement, even though it cannot serve as the primary imaging modality for this procedure.

Transesophageal echocardiography serves several important functions during Impella RP insertion. First, it allows identification of any cause of hemodynamic instability during device placement that might result from mechanical interference of the catheter with the tricuspid or pulmonary valve apparatus or direct trauma to the RV free wall. The relatively thin wall of the right ventricle makes it vulnerable to perforation, and real-time echocardiographic monitoring can detect early signs of pericardial effusion that might indicate this catastrophic complication. TEE also facilitates early identification of any significant pericardial effusion, whether from procedural complications or pre-existing disease, allowing prompt intervention before hemodynamic compromise develops.

The anatomical landmarks of the Impella RP device can be identified by chest fluoroscopy, with the inlet positioned in the inferior vena cava and the outlet in the distal main pulmonary artery, targeting a position optimally 2–4 cm above the pulmonic valve plane as recommended by the manufacturer and supported by case-series data [20]. Optimal outlet distance has not been evaluated in controlled studies.

Fluoroscopy allows simultaneous assessment of both the inlet and outlet markers and their spatial relationship, with the ideal configuration described as a “north-south” orientation in a non-dilated right ventricle [20]. In contrast to fluoroscopy, TEE cannot visualize both the inflow and outflow portions of the device with a single imaging plane due to the complex three-dimensional trajectory of the catheter through the right heart.

The most suitable TEE views for Impella RP assessment include: the mid-esophageal bicaval projection (90–110°) for visualizing the inflow portion of the device in the inferior vena cava and confirming its relationship to the caval-atrial junction; the mid-esophageal four-chamber view (0°) with rightward rotation and focus on the RV, which allows visualization of the cannula as it passes through the tricuspid valve and into the right ventricular chamber; and the mid-esophageal short-axis RV inflow-outflow view (45°), which permits assessment of the Impella RP course as it traverses the tricuspid valve, passes through the RV, and exits across the pulmonic valve, with confirmation that the outflow portion is positioned 2–4 cm above the valve plane.

Following device placement, left ventricular function must be carefully evaluated, as the hemodynamic effects of effective RV unloading include increased pulmonary blood flow and consequently increased preload to the left ventricle. A left ventricle with previously unrecognized or borderline dysfunction may become overloaded under these conditions, potentially precipitating pulmonary edema or LV failure. Additionally, adequate native LV cardiac output is essential for proper Impella RP pump function, as the device depends on blood returning from the systemic circulation to maintain adequate inlet flow. Point-of-care chest ultrasonography is also valuable in this setting for assessing pulmonary congestion by evaluating B-line patterns and pleural effusions.

Echocardiographic evaluation remains crucial throughout the support period for assessing the right ventricle’s response to mechanical unloading. Signs of improving RV function—including decreasing RV size, improving TAPSE and S′ velocity, and normalization of septal motion—suggest successful support and potential for recovery. The correct position of the device should be evaluated frequently, as RV remodeling during the recovery process may cause device displacement requiring repositioning.

**Figure 6:**
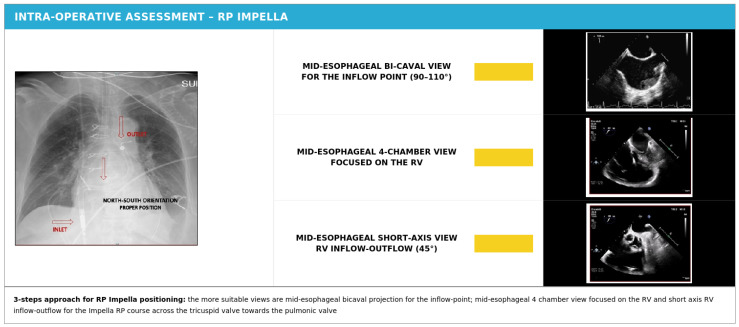
Main views for intra-operative echocardiographic assessment for RP Impella^®^ positioning.

Transthoracic echocardiography (TTE) can also provide valuable information in the postoperative setting and has the advantage of being non-invasive and repeatable at the bedside. The transthoracic apical four-chamber view allows serial evaluation of RV volume, basal and mid-cavity diameters, and longitudinal function parameters (TAPSE and tissue Doppler S′ velocity). The parasternal long-axis view can be used to estimate RV outflow tract (RVOT) diameter, while the parasternal short-axis view at the level of the great vessels allows measurement of both proximal and distal RVOT diameters. The subcostal projection is particularly useful for visualizing the device’s inflow portion in the inferior vena cava and for detecting pericardial effusion [21]. However, TEE generally remains more suitable than TTE for detailed evaluation of the tricuspid and pulmonary valves due to their anatomic positions and the significant Doppler artifacts generated by the functioning Impella RP device (Figure 6 and Table 3).

## 6. Postoperative Assessment of Left-Sided Impella

The optimal position and function of left-sided Impella devices depend on several essential factors that require ongoing echocardiographic surveillance throughout the support period. First and foremost, the insertion depth of the device into the ventricle is crucial for optimizing the pump’s unloading effect. As previously noted, the pump inlet should be positioned at 3.5–4 cm below the aortic valve plane for the Impella CP and approximately 5.5 cm for the Impella 5.5. Device position can change during the support period due to patient movement, changes in ventricular geometry, or inadvertent catheter migration, necessitating regular echocardiographic verification.

The second critically important element is the rotational axis of the device—its three-dimensional orientation within the left ventricular cavity. The ideal orientation directs the inlet area toward the ventricular apex, with the concavity of the curved catheter facing the interventricular septum. This positioning ensures unobstructed inflow from the blood pool at the ventricular apex and minimizes the risk of the inlet becoming positioned against the ventricular wall or mitral valve apparatus.

Impella malrotation is a clinically significant complication defined by a characteristic combination of echocardiographic findings and abnormal device-derived parameters. The clinical syndrome is characterized by abnormal pressure waveforms and motor current tracings displayed on the Impella controller console, incorrect device depth across the aortic valve relative to manufacturer recommendations, and abnormal orientation of the pigtail, which is directed toward the lateral LV wall rather than toward the ventricular apex.

**Figure 7:**
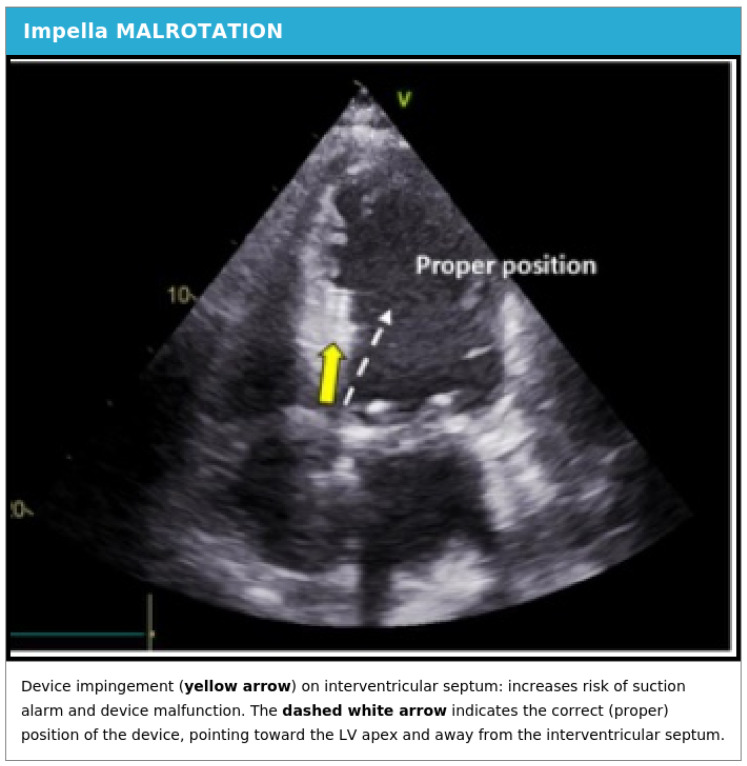
Echocardiographic evaluation of Impella malrotation.

In the setting of malrotation, the concavity of the Impella catheter does not face the interventricular septum as intended but instead is directed toward the lateral wall or mitral valve apparatus. This abnormal orientation frequently leads to mechanical impingement on the mitral subvalvular apparatus and positioning of the inlet area in close proximity to the mitral valve leaflets. The clinical consequences of these malpositions can be severe and include flow obstruction at the device inlet (causing suction alarms and reduced device output), mechanical hemolysis from turbulent flow and cell trauma, and reduced unloading efficiency that fails to achieve the intended hemodynamic goals. Representative echocardiographic findings of Impella malrotation are demonstrated in Figure 7.

The clinical significance of Impella malposition should not be underestimated. Published data from a single-centre retrospective study by Baldetti et al. indicate that malposition was observed in up to 40% of patients with cardiogenic shock, making it potentially one of the most common device-related complications. The generalisability of this figure to other settings remains to be confirmed. Importantly, malposition has been associated with suboptimal left ventricular unloading, higher indices of both pulsatile and steady components of right ventricular afterload, and significantly worse serum lactate clearance from patient admission through the subsequent 48-h period. These findings underscore the importance of vigilant echocardiographic monitoring and prompt intervention when malposition is detected [22].

The primary aim of postoperative echocardiographic surveillance is to identify suboptimal device positioning early, before the patient experiences the detrimental hemodynamic and hematologic consequences of malrotation, and to guide catheter repositioning maneuvers. The relationship between preoperative anatomical features and the propensity for malrotation remains an area of active investigation. A narrow mitral-aortic angle (the angle between the planes of the mitral and aortic valve annuli) and the presence of a hypertrophied left ventricle with dynamic outflow tract obstruction have been suggested as anatomical conditions that may predispose to device malrotation [23].

Once the patient demonstrates clinical evidence of improved systemic perfusion, a structured weaning strategy should be initiated and guided by comprehensive multiparametric evaluation in which echocardiography plays an indispensable role. The assessment of native cardiac function during Impella support presents unique challenges, as the presence of the device itself significantly complicates the standard echocardiographic evaluation techniques.

**Figure 8:**
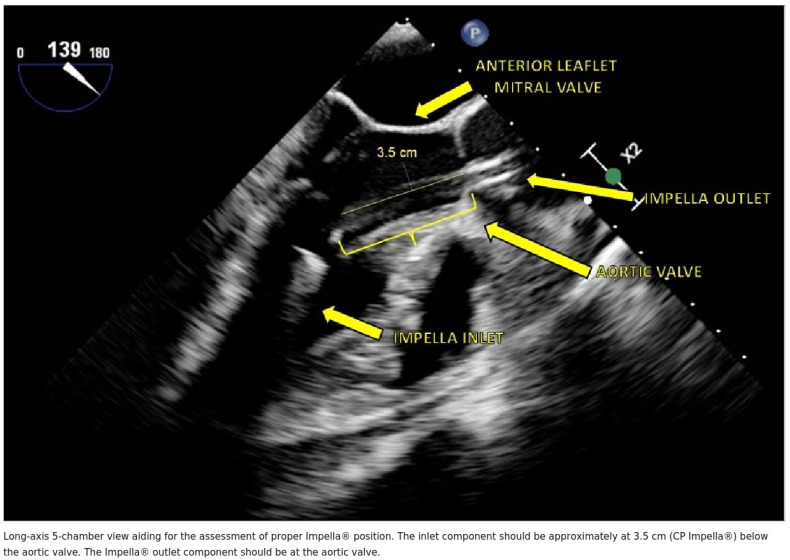
Proper position of left side Impella^®^.

**Figure 9:**
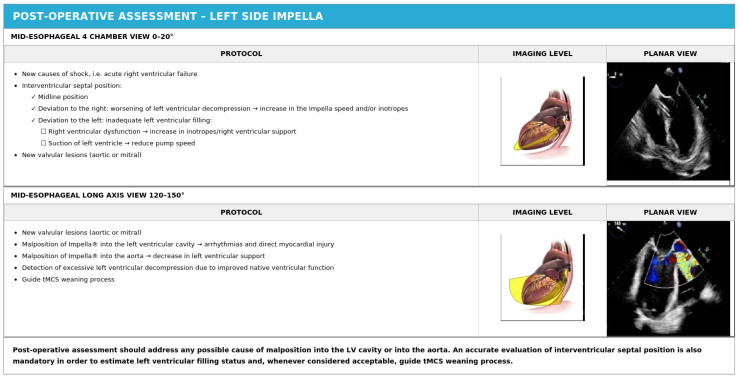
Post-operative echocardiographic assessment for Impella^®^ positioning.

In Impella-supported patients, the most commonly used Doppler-based measurements of cardiac output may be significantly affected by the mechanical noise generated by the rotating impeller and the continuous flow characteristics of the device. The LVOT velocity–time integral (VTI), which is routinely used to estimate stroke volume and cardiac output in non-supported patients, may be unreliable during Impella support because the continuous forward flow generated by the device is superimposed on any native cardiac output, making it impossible to separate the two contributions. Furthermore, the standard biplane method for calculating left ventricular ejection fraction may be insufficient for detecting early myocardial recovery or for accurately guiding weaning decisions in patients with severe baseline LV dysfunction [24]. The proper position and key elements of postoperative assessment of left-sided Impella devices are illustrated in Figure 8 and Figure 9, Table 4.

## 7. Postoperative Assessment of the Impella RP

The comprehensive postoperative evaluation of patients supported with Impella RP requires an integrated multiparametric approach that combines invasive hemodynamic monitoring data with serial echocardiographic assessments. This combined approach is essential for optimizing device function, detecting complications, assessing the response to mechanical unloading, and determining the appropriate timing for device weaning and removal.

Transesophageal echocardiography should be used to accurately and systematically investigate right ventricular function and its response to mechanical support. Key parameters include tricuspid annular plane systolic excursion (TAPSE), which provides a reproducible measure of RV longitudinal function; RV imaging at the lateral tricuspid annulus, which yields the S′ velocity; and serial measurement of RV end-diastolic diameter to assess changes in chamber size. Improvement in these parameters during support suggests successful RV unloading and potential for recovery, while failure to improve or worsening values may indicate inadequate support, device malposition, or irreversible RV injury.

Concurrent evaluation of left ventricular function is equally essential during Impella RP support, as the presence of right ventricular dysfunction may mask or underestimate coexisting LV dysfunction by limiting preload to the left heart. As RV function improves with mechanical unloading and pulmonary blood flow increases, previously occult LV dysfunction may become clinically apparent. Serial echocardiographic assessment of LV size and function allows early detection of this phenomenon and appropriate adjustment of the treatment strategy.

As treatment of RV failure progresses beyond the initial stabilization phase, pump displacement and the potential need for device repositioning become increasingly important concerns that must be systematically addressed. The management of these technical challenges depends not only on the accuracy of the original device positioning but also on the dynamic changes in RV shape and dimensions that occur as the ventricle responds to unloading. RV geometry varies considerably from patient to patient at baseline and can change dramatically during the support period as remodeling occurs.

**Figure 10:**
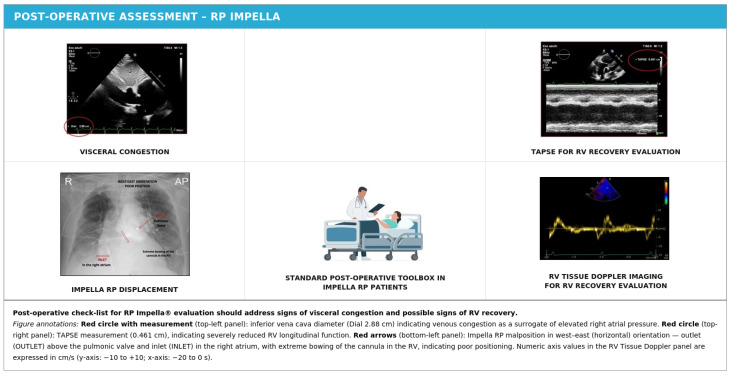
Standard post-operative check-list for RP Impella^®^ evaluation.

Although systematic data on this phenomenon are limited, clinical experience suggests that RV remodeling—particularly when occurring on a chronic basis prior to device implantation—is a complex and multifaceted process that significantly affects device stability. The shape and dimensions of the right ventricle may change substantially during the support period, potentially causing the device to migrate from its optimal position. In patients with particularly severe RV dilation and adverse remodeling, the Impella RP may be more technically challenging to implant initially and more difficult to maintain in the correct position throughout the support period, potentially requiring more frequent echocardiographic monitoring and repositioning maneuvers [5,20]. These considerations are illustrated in Figure 10 and Table 4.

## 8. Complications and Troubleshooting Under Echocardiographic Guidance

Despite the substantial and well-documented clinical benefits of Impella devices in appropriately selected patients, their use is not without potential complications. Many of these complications can be promptly identified and effectively managed through systematic echocardiographic evaluation, underscoring the importance of imaging expertise in centers using these devices. Echocardiography provides essential real-time information on device position relative to cardiac structures, changes in ventricular chamber dimensions, and valve function, thereby enabling early detection of adverse events and optimization of the mechanical support strategy.

Device malposition represents one of the most frequent complications encountered during Impella support and may occur either during the initial insertion procedure or subsequently during the support period. Causes of delayed malposition include patient movement and repositioning in bed, changes in cardiac chamber dimensions as the ventricle responds to unloading, progression or recovery of underlying cardiac pathology, and inadvertent catheter migration during routine patient care activities. Both TEE and TTE can effectively identify abnormal device orientation, including positioning of the inlet away from the LV apex, device impingement against the ventricular septum, and mechanical interference with the mitral sub-valvular apparatus. Prompt recognition and correction of malposition is essential to prevent downstream complications including suction events (which trigger device alarms and reduce output) and mechanical hemolysis—both of which can result in worsening hemodynamic instability and end-organ dysfunction [19].

Hemolysis remains a significant clinical issue in patients receiving prolonged Impella support and represents an important cause of morbidity. Hemolysis results primarily from mechanical shear stress on red blood cells as they pass through the rotating impeller, and this effect is exacerbated when the device inlet or outlet is partially obstructed or when suction events occur due to excessive unloading relative to ventricular preload. Echocardiographic findings that suggest hemolysis-promoting conditions include a collapsed or underfilled left ventricle with minimal or absent opening of the aortic valve (indicating inadequate preload), and color Doppler evidence of turbulent or disturbed flow patterns near the device inlet (suggesting partial obstruction). Management strategies for hemolysis include optimization of pump position through catheter repositioning, reduction of pump speed to decrease shear forces, and volume resuscitation to improve ventricular filling when appropriate [25].

Importantly, volume resuscitation to improve ventricular filling must be undertaken with extreme caution in patients with concomitant or predominant right ventricular dysfunction. In the setting of severe RV failure, excessive fluid administration can lead to further RV dilatation, worsening of functional tricuspid regurgitation, and leftward interventricular septal shift (D-shaped left ventricle), ultimately compromising LV filling and reducing systemic cardiac output. Echocardiographic monitoring during volume challenge is therefore essential: serial assessment of RV dimensions, septal position (eccentricity index), and tricuspid regurgitation severity should guide fluid management. If echocardiographic signs of RV overload—progressive RV dilatation, worsening septal bowing, increasing tricuspid regurgitation—are observed during volume administration, fluid resuscitation should be promptly discontinued and vasopressor or inotropic support should be considered as an alternative strategy to optimize preload and RV contractility.

Valvular injury represents another important potential complication of Impella therapy that can result from device malalignment, traumatic insertion, or prolonged mechanical impingement on valve structures. Transesophageal echocardiography is highly sensitive for detecting new-onset valvular regurgitation or leaflet restriction that may indicate valve injury. Early identification of valve dysfunction allows prompt device repositioning or removal before irreversible structural damage develops. It is important to recognize that pre-existing valve disease—particularly aortic regurgitation—may worsen under the hemodynamic stress of device support, further emphasizing the importance of thorough pre-implantation valve assessment [26].

Left ventricular thrombus formation is another recognized complication that can occur during Impella support, particularly in the setting of low-flow states, severely reduced ventricular contractility, or prolonged support duration. The presence of a foreign body (the Impella catheter) within the ventricular cavity may serve as a nidus for thrombus formation, particularly when combined with blood stasis. Echocardiography can identify thrombus as echogenic masses, typically located near the ventricular apex or adherent to the pigtail portion of the device. These findings are often associated with spontaneous echo contrast within the ventricular cavity. Detection of ventricular thrombus requires intensification of anticoagulation therapy and careful adjustment of pump parameters to optimize blood flow and prevent embolic complications.

Pericardial effusion and cardiac tamponade represent potentially life-threatening complications that may develop either from direct procedural complications during device insertion or from myocardial perforation by the guidewire or device catheter. The accumulating pericardial fluid progressively increases intrapericardial pressure, which restricts right ventricular filling and reduces preload to both ventricles. Consequently, forward flow through the Impella may decrease progressively, often manifesting as declining device output even before overt hypotension and clinical shock develop. Echocardiography is the diagnostic modality of choice for this complication, allowing immediate identification of pericardial fluid accumulation and detection of the hemodynamically significant signs of tamponade physiology, including right atrial and right ventricular diastolic collapse [27].

For right-sided Impella RP devices, malposition is similarly among the most common technical complications encountered. The unique anatomy and trajectory of the RP device create specific vulnerabilities: the inlet portion may migrate proximally into the right atrium, reducing effective aspiration of blood, or the outlet may migrate distally into the peripheral pulmonary arteries or proximally toward the pulmonic valve, in either case compromising effective forward flow. Echocardiography, particularly TEE, allows real-time visualization of the device inlet position, assessment of RV wall contact that might cause suction or injury, and detection of tricuspid valve leaflet entrapment that could cause regurgitation or obstruction. Malposition of the Impella RP can result in suction events, inadequate ventricular unloading with persistent RV failure, or mechanical trauma to the RV free wall or tricuspid valve apparatus. Prompt detection and repositioning are essential to prevent progressive hemodynamic deterioration [28].

Published observational data suggest that device-related complications are common during Impella support: malposition has been reported in up to 40% of patients in a single-centre cohort (Baldetti et al.), haemolysis occurs in approximately 5–10% of cases, and suction events may arise whenever left ventricular preload is inadequate. Given the frequency and potential severity of these complications, regular and systematic echocardiographic surveillance is strongly recommended for all patients receiving Impella support in the intensive care unit setting, consistent with emerging expert consensus [18,19].

Standardized monitoring protocols, including scheduled daily echocardiographic assessments and immediate imaging in response to any clinical or device-derived evidence of malfunction, should be implemented at all centers using these devices.

## 9. Weaning Strategies and Echocardiographic Assessment

The decision to reduce or discontinue Impella mechanical circulatory support represents a critical juncture in patient management that should be based on a comprehensive, multiparametric evaluation integrating hemodynamic data, biochemical markers of end-organ function, and echocardiographic parameters of cardiac recovery. This integrated approach aims to accurately identify true myocardial recovery and readiness for device removal while minimizing the risk of premature device weaning, which could precipitate recurrent cardiogenic shock, the need for emergency re-escalation of mechanical support, or death [29].

For patients supported with a left-sided Impella device, the weaning process is typically performed by gradually reducing the pump speed in a stepwise fashion, commonly referred to as P-level reduction. This gradual approach provides the clinical team with the opportunity to carefully observe the native left ventricle’s response to progressively increasing workload as mechanical support is withdrawn. Throughout this process, continuous hemodynamic monitoring and serial echocardiographic assessments should guide decision-making.

Several specific echocardiographic signs suggest that the heart may be sufficiently recovered to assume full responsibility for maintaining systemic circulation without mechanical support. The reappearance of aortic valve opening during device support—or its persistence at lower support levels—indicates that the native left ventricle is generating sufficient systolic pressure to overcome aortic impedance and eject blood forward. This finding suggests preservation or recovery of contractile reserve. Similarly, an increase in the LVOT velocity–time integral to greater than 10–12 cm during reduced support, and improvement in left ventricular ejection fraction to above 35%, has been proposed as markers of meaningful recovery in observational studies and expert weaning protocols [24,29,30]. These cut-offs are based on limited, mostly single-centre data (Level C evidence) and have not been validated in randomised weaning trials. Clinical decision-making should integrate these values within the broader haemodynamic and biochemical context of each patient.

Assessment of mitral valve dynamics and diastolic function also provides important prognostic information during the weaning process. A reduction in the severity of functional mitral regurgitation during weaning suggests improved LV geometry and papillary muscle function. Normalization of diastolic filling pressures, as estimated through non-invasive indices such as the E/e′ ratio, indicates more efficient ventricular-atrial coupling and improved diastolic relaxation and compliance. Together, these findings suggest recovery of both systolic and diastolic ventricular function.

Conversely, certain echocardiographic findings during the weaning trial should prompt caution and consideration of delaying further weaning attempts. Persistence of left ventricular dilation despite adequate support duration, sustained elevation of filling pressures during weaning attempts, or reduction in pulsatility index all suggest incomplete myocardial recovery and warn of potential weaning failure. In these situations, allowing additional time for myocardial rest and recovery before reattempting weaning is essential to avoid precipitating hemodynamic deterioration [30].

An important practical consideration during the weaning process is that excessive diuresis—often employed in an attempt to optimize hemodynamics and facilitate weaning—can paradoxically increase the risk of complications. Aggressive diuresis leading to hypovolemia and a small, underfilled LV cavity increases the risk of device suction events, which trigger alarms, cause hemolysis, and may damage ventricular structures. Careful attention to volume status and preload optimization is therefore essential throughout the weaning process.

When considering weaning from right-sided support with Impella RP, the clinical focus appropriately shifts to assessment of right ventricular function and reserve. The right ventricle has unique physiological characteristics—including a thin wall, high compliance, and marked sensitivity to changes in preload and afterload—that make accurate assessment of recovery particularly challenging. Echocardiographic markers suggesting adequate RV recovery and readiness for device weaning include TAPSE greater than 15 mm, tissue Doppler S′ velocity above 10 cm/s, and RV fractional area change greater than 35%. These thresholds have been extrapolated from general RV failure literature and expert opinion; to date, no dedicated studies have prospectively validated specific echocardiographic cut-offs for Impella RP weaning readiness.

Assessment of RV geometry and interventricular septal motion provides additional important information during RP weaning. A midline interventricular septum, or one that appropriately shifts leftward during weaning trials, indicates restored normal RV–LV interaction and balanced filling dynamics between the two ventricles. Conversely, persistent septal flattening or paradoxical septal motion during weaning suggests ongoing RV pressure or volume overload and incomplete recovery.

The ability to maintain stable pulmonary artery pressures despite stepwise reductions in Impella RP support represents a key indicator of successful recovery. This finding indicates that the right ventricle is capable of independently generating and sustaining adequate forward flow into the pulmonary circulation without continued reliance on mechanical unloading. In contrast, a drop in pulmonary artery pressures during weaning attempts may signal inadequate native RV output and persistent dependence on device support, warranting continued therapy.

RV function is generally more fragile and unpredictable during weaning compared to LV function due to the inherent characteristics of the right ventricle. The thin-walled RV, with its high compliance and marked dependence on adequate preload and low afterload, can be destabilized by relatively small changes in venous return or pulmonary vascular resistance. These sensitivities can significantly affect RV output and predispose to hemodynamic instability during the gradual reduction of Impella RP support [31].

Even after successful Impella explantation, echocardiography retains a critically important role in post-removal surveillance. Serial imaging studies performed at 24–48 h following device removal and again before hospital discharge serve to identify potential late complications that may not be immediately apparent. These complications include acquired aortic valve dysfunction related to prolonged commissural immobilization by the device, which may manifest as new or worsening aortic regurgitation; residual or newly formed ventricular thrombus at the previous device location; and evolving regional wall motion abnormalities or global dysfunction that were not evident during mechanical support.

In selected patients—particularly those with uncertain recovery trajectories or clinical scenarios that do not conform to typical patterns—cardiac magnetic resonance imaging (CMR) can provide valuable complementary information to echocardiography. CMR offers high-resolution tissue characterization capabilities including quantification of myocardial edema (suggesting ongoing inflammation or injury), detection and quantification of myocardial necrosis and scar, and assessment of diffuse myocardial fibrosis. CMR-derived strain parameters and tissue characterization markers offer deeper pathophysiological insight into myocardial viability and long-term remodeling potential, helping to refine prognostic assessment and guide subsequent management strategies [32] (Table 5).

## 10. Conclusions

Impella percutaneous ventricular assist devices have become an integral component of cardiogenic shock management, with the DanGer Shock trial providing the first randomised evidence of mortality benefit in AMI-related cardiogenic shock. However, the success of Impella therapy depends critically on accurate patient selection, precise device positioning, and vigilant monitoring throughout the support period.

Echocardiography is the cornerstone imaging modality across all phases of Impella therapy. Its real-time capabilities allow clinicians to assess candidacy and anatomy before implantation, guide device positioning intraoperatively, monitor performance and detect complications during support, and evaluate recovery to guide weaning. The development of standardised echocardiographic protocols, specifying required views, key measurements, and evidence-based thresholds for each phase, is essential for ensuring safety and optimising outcomes across institutions.

It is important to acknowledge that the majority of echocardiographic thresholds and protocols discussed in this review are derived from observational data, expert consensus, and manufacturer recommendations rather than from randomised trials. Multicentre registries and collaborative research networks are needed to prospectively validate echocardiographic-guided algorithms for patient selection, device optimisation, and weaning decision-making, ultimately contributing to the development of formal clinical practice guidelines for this rapidly evolving field. The main steps for echocardiographic evaluation in Impella device implantation and management are summarised in the Central Illustration.

Highlights

- The Impella device represents an important therapeutic tool in the contemporary management of cardiogenic shock, with recent evidence demonstrating mortality benefit in appropriately selected patients.
- Echocardiographic evaluation is essential and strongly recommended throughout all phases of Impella therapy, including pre-implantation assessment, procedural guidance for device positioning, postoperative monitoring, complication detection, and weaning decision-making.
- Device malposition occurs in up to 40% of cardiogenic shock patients and is associated with suboptimal ventricular unloading and worse clinical outcomes, emphasizing the importance of vigilant echocardiographic surveillance.
- Specialized training in device-specific echocardiographic assessment and implementation of standardized imaging protocols are essential for optimizing outcomes in centers using Impella devices.

## Figures and Tables

**Table 1:** Impella Devices.

	Impella 5.5 SmartAssist	Impella CP	Impella CP SmartAssist	RP Impella
**Catheter size**	9 Fr			
**Maximum average** **flow**	6 L/min	4.3 L/min		>3.5 L/min
**Placement sensor**	Fiber optic sensor	Pressure sensor	Fiber optic sensor	
**Bore sheat** **dimensions**	21 Fr	14 Fr	14 Fr	9 Fr
**Cannula**	Curved	Curved, Pigtail		Curved, Pigtail
**Access site**	**Surgical** Aortic or axillary	**Percutaneous** Femoral (Alternative: axillary)		
**Wire-guide** **diameter**	0.0018″			
**Average length of** **support**	30 days	5 days		

The Impella family of devices are short-term mechanical circulatory support (MCS) pumps designed to provide hemodynamic support in case of insufficient native cardiac output. Left Impella devices are different in size and flow. Only one device is available for RV support.

**Table 2:** Echocardiographic Assessment of Impella Devices. Pre-Implantation Assessment.

Category	Left-Sided Impella (CP/5.5)	Impella RP
**Recommended** **Echo Views**	ME AV LAX & SAX (aortic valve morphology) ME 4-chamber & 2-chamber (LV size, EF, WMA, thrombus) TG SAX (circumferential contraction) ME RV inflow-outflow & 4-ch (RV function) ME ascending aortic LAX; descending aortic SAX/LAX TTE parasternal & apical views (bedside pre-screening)	ME 4-chamber (biventricular size & function) ME RV inflow-outflow (TV/PV morphology) TG RV inflow (RV geometry, thrombus) ME bicaval (IVC, RA thrombus) TTE apical 4-ch, subcostal (serial RV assessment)
**Key** **Measurements**	LVEF (biplane), LV EDD AV regurgitation/stenosis grade LVOT VTI (<10 cm = poor native output) RV/LV basal diameter ratio (>1.0 abnormal) LV eccentricity index (>1.1 = RV pressure overload) TAPSE (>17 mm), S′ (>9.5 cm/s), RVFAC (>35%) AV opening status	TAPSE, S′, RVFAC RV basal & mid-cavity diameters Septal position & eccentricity index TR & PR severity LV function (EF, LV EDD)—rule out biventricular failure
**Absolute** **Contraindications**	Moderate-to-severe aortic regurgitation Severe aortic stenosis Mechanical aortic prosthesis LV thrombus Aortic dissection/severe aortic atheroma	Mechanical tricuspid or pulmonary prosthesis Moderate-severe TV or PV stenosis RA/IVC mural thrombus
**Relative Contra-** **indications/Flags**	Severe biventricular failure (LV unloading may precipitate RV failure) Papillary muscle rupture, VSD, tamponade (alternative Rx first)	Severe TR (relative; may improve post-unloading) Severe RV dilatation/remodelling (positioning challenges) Unrecognised LV dysfunction (risk of LV overload with RP)

**ECPella setting:** On VA-ECMO, absent AV opening + LVOT VTI < 10 cm + LV EDD > 6.1 cm + dense spontaneous echo contrast → strong indication for adding Impella for LV unloading.

**Table 3:** Intraoperative (During Implantation).

Category	Left-Sided Impella (CP/5.5)	Impella RP
**Recommended** **Echo Views**	Primary: ME AV LAX (guidewire → catheter → final position) ME AV SAX (catheter crossing valve) Colour-flow Doppler across inlet & outlet	Primary: Fluoroscopy (mandatory for RP) ME bicaval 90–110° (inlet in IVC) ME 4-ch with rightward rotation (cannula through TV) ME SAX RV inflow-outflow 45° (outlet above PV)
**Key Targets**	Inlet depth below AV plane: CP 3.5–4 cm; 5.5: 4–5 cm Outlet ~1.5 cm above AV plane Inlet directed toward LV apex, between papillary muscles Catheter concavity facing interventricular septum ⚠ Do NOT include pigtail (CP only) in depth measurement	Inlet in IVC (below caval-atrial junction) Outlet 2–4 cm above pulmonic valve plane “North-south” orientation on fluoroscopy in non-dilated RV
**Signs of** **Malposition**	Guidewire entangled with papillary muscles/chordae Catheter impinging on MV leaflets → acute MR Inlet abutting septum → suction risk New/worsening AR after catheter passage → valve injury	Catheter looping in RA or RV New pericardial effusion (thin RV wall → perforation risk) TV leaflet entrapment → acute TR Haemodynamic instability during passage
**Corrective** **Actions**	Reposition guidewire if entangled before advancing catheter Pull back/re-advance catheter if MV or septal contact Reassess AV leaflets post-placement; reposition if new AR Confirm position in multiple planes (curved trajectory)	Fluoroscopic repositioning under TEE haemodynamic surveillance Immediate pericardiocentesis if effusion + tamponade physiology Re-evaluate LV function after effective RV unloading (risk of LV overload)

**Table 4:** Post-Implantation Monitoring (ICU).

Category	Left-Sided Impella (CP/5.5)	Impella RP
**Recommended Echo Views**	ME LAX (device depth, rotational axis, valve interaction) ME 4-ch/2-ch (LV size, EF, thrombus, SEC) Colour Doppler at inlet & outlet TTE parasternal & apical (daily bedside)	ME bicaval (inlet position in IVC) ME 4-ch (RV size, TAPSE, septal motion) ME SAX RV inflow-outflow (outlet position above PV) TTE apical 4-ch, subcostal (serial, non-invasive) Lung ultrasound (B-lines, pleural effusion)
**Key Measurements**	Device depth below AV (verify daily; migration common) Pigtail orientation (should point toward apex) AV opening (absent = LV not ejecting) LV dimensions (distension = inadequate unloading) MR severity (new/worsening = device interference)	TAPSE, S′, RVFAC (trend for recovery) RV end-diastolic diameter (shrinking = good response) Septal position (flattening = persistent RV overload) LV function (unmask LV dysfunction as RV improves) Inlet/outlet position (RV remodelling → displacement)
**Warning Signals**	Malrotation (up to 40%): concavity facing lateral wall; pigtail toward lateral wall; abnormal console waveforms/motor current Suction events: inlet against septum or wall; collapsed/underfilled LV; console alarms Hemolysis: turbulent colour Doppler at inlet; partially obstructed inlet/outlet; small LV cavity Thrombus: echogenic mass near apex or on pigtail; dense SEC Tamponade: new pericardial effusion; RA/RV diastolic collapse; declining device output Valve injury: new AR or worsening MR post-placement	Inlet migration into RA → reduced aspiration Outlet migration into distal PA or toward PV → poor forward flow TV leaflet entrapment → worsening TR RV wall contact → suction, possible perforation LV failure unmasked by increased pulmonary flow Pericardial effusion (RV perforation)
**Corrective Manoeuvres**	Catheter repositioning (depth & rotation) under echo guidance Reduce pump speed if suction events/hemolysis Volume resuscitation if underfilled LV Intensify anticoagulation if thrombus/SEC Urgent pericardiocentesis if tamponade Consider device removal if irreversible valve injury	Fluoroscopic repositioning with echo monitoring Increase echo surveillance frequency if severe RV dilatation Add LV support if LV failure unmasked (BiPella or ECPella) Pericardiocentesis/surgical repair if perforation

**Risk factors for left-sided malrotation:** narrow mitral-aortic angle; hypertrophied LV with dynamic LVOT obstruction. Consider more frequent surveillance in these patients. Dark navy header—Standard table header styling for title and column names. Teal font/light blue background (Recommended Echo Views; Key Measurements)—Informational and procedural categories. Red font/light red background (Warning Signals; Corrective Manoeuvres)—Safety-critical categories; consistent with clinical warning conventions.

**Table 5:** Weaning & Post-Explantation.

Category	Left-Sided Impella (CP/5.5)	Impella RP
**Protocol**	Stepwise P-level reduction with continuous haemodynamic & echo monitoring	Gradual pump speed reduction with serial RV assessment
**Echo Views**	ME LAX (AV opening, LVOT VTI) ME 4-ch/2-ch (LVEF, LV dimensions) Pulsed-wave Doppler at MV (E/e′ ratio) Colour Doppler (MR severity trend)	ME/TTE 4-ch (TAPSE, S′, RV dimensions) TG SAX/parasternal SAX (septal motion) PA pressure estimation (TR jet velocity)
**Signs of Recovery (proceed with weaning)**	AV opening reappears/persists at lower support LVOT VTI > 10–12 cm LVEF > 35% Decreasing functional MR Normalising E/e′ ratio (improved diastolic function)	TAPSE > 15 mm S′ > 10 cm/s RVFAC > 35% Midline or leftward septal shift during wean trial Stable PA pressures despite reduced support
**Signs of Failed Weaning (delay/abort)**	Persistent LV dilatation Sustained elevated filling pressures (high E/e′) Reduced pulsatility index Worsening MR during wean trial ⚠ Excessive diuresis → hypovolaemia → suction events & hemolysis	Persistent septal flattening/paradoxical motion Drop in PA pressures (inadequate native RV output) RV re-dilatation Declining TAPSE/S′
**Post-Explant Surveillance**	Echo at 24–48 h post-removal and again before discharge Assess for: new/worsening AR (commissural immobilisation), residual or new LV thrombus, evolving WMA or global dysfunction Consider CMR in uncertain recovery: myocardial oedema, necrosis/scar quantification, diffuse fibrosis, strain analysis	

Dark navy column headers—Standard column header styling. Teal font/light blue background (Echo Views; Signs of Recovery)—Informational and procedural categories. Red font/light red background (Signs of Failed Weaning)—Safety-critical categories indicating potential clinical deterioration; consistent with clinical warning conventions. Grey font/light grey background (Protocol; Post-Explant Surveillance)—General procedural guidance applicable to both device types. Abbreviations: AR, aortic regurgitation; AV, aortic valve; CMR, cardiac magnetic resonance; E/e′, ratio of early mitral inflow velocity (E) to mitral annular early diastolic velocity (e′); LVEF, left ventricular ejection fraction; LV, left ventricle; LVOT VTI, left ventricular outflow tract velocity-time integral; ME, mid-esophageal; ME 4-ch/2-ch, mid-esophageal 4-chamber/2-chamber view; ME LAX, mid-esophageal long-axis view; MR, mitral regurgitation; MV, mitral valve; PA, pulmonary artery; P-level, Impella pump performance level (P0–P9); RV, right ventricle; RVFAC, right ventricular fractional area change; S′, tricuspid annular peak systolic velocity (tissue Doppler imaging); SAX, short-axis view; TAPSE, tricuspid annular plane systolic excursion; TG SAX, trans-gastric short-axis view; TR, tricuspid regurgitation; TTE, transthoracic echocardiography; WMA, wall motion abnormalities; ⚠, caution/clinically important warning point.

## Data Availability

No new data were created or analyzed in this study.

## Abbreviations

CMR, cardiac magnetic resonance; CS, cardiogenic shock; EDD, end-diastolic diameter; LAX, long axis; LV, left ventricle; LVEF, left ventricular ejection fraction; LVOT, left ventricular outflow tract; MCS, mechanical circulatory support; RV, right ventricle; RVFAC, right ventricular fractional area change; RVOT, right ventricular outflow tract; SAX, short axis; SCAI, Society for Cardiovascular Angiography and Interventions; TAPSE, tricuspid annular plane systolic excursion; TEE, transesophageal echocardiography; TTE, transthoracic echocardiography; VA-ECMO, veno-arterial extracorporeal membrane oxygenation; VTI, velocity–time integral.
